# Pan-*Plasmodium* band sensitivity for *Plasmodium falciparum* detection in combination malaria rapid diagnostic tests and implications for clinical management

**DOI:** 10.1186/s12936-015-0629-z

**Published:** 2015-03-18

**Authors:** Michelle L Gatton, Roxanne R Rees-Channer, Jeffrey Glenn, John W Barnwell, Qin Cheng, Peter L Chiodini, Sandra Incardona, Iveth J González, Jane Cunningham

**Affiliations:** School of Public Health and Social Work, Queensland University of Technology, GPO Box 2434, Brisbane, Qld 4001 Australia; National Institute for Health Research University College London Hospitals Biomedical Research Centre, Hospital for Tropical Diseases, London, UK; Malaria Branch, Division of Parasitic Diseases and Malaria, Centers for Disease Control and Prevention, Center for Global Health, Atlanta, USA; Drug Resistance and Diagnostics, Australian Army Malaria Institute, Brisbane, Australia; London School of Hygiene and Tropical Medicine, London, UK; FIND (Foundation for Innovative New Diagnostics), Geneva, Switzerland; Global Malaria Programme, World Health Organization, Geneva, Switzerland

**Keywords:** Malaria, Rapid diagnostic test, HRP2, pLDH, Persistent antigenemia

## Abstract

**Background:**

Malaria rapid diagnostic tests (RDTs) are appropriate for case management, but persistent antigenaemia is a concern for HRP2-detecting RDTs in endemic areas. It has been suggested that pan-pLDH test bands on combination RDTs could be used to distinguish persistent antigenaemia from active *Plasmodium falciparum* infection, however this assumes all active infections produce positive results on both bands of RDTs, an assertion that has not been demonstrated.

**Methods:**

In this study, data generated during the WHO-FIND product testing programme for malaria RDTs was reviewed to investigate the reactivity of individual test bands against *P. falciparum* in 18 combination RDTs. Each product was tested against multiple wild-type *P. falciparum* only samples. Antigen levels were measured by quantitative ELISA for HRP2, pLDH and aldolase.

**Results:**

When tested against *P. falciparum* samples at 200 parasites/μL, 92% of RDTs were positive; 57% of these on both the *P. falciparum* and pan bands, while 43% were positive on the *P. falciparum* band only. There was a relationship between antigen concentration and band positivity; ≥4 ng/mL of HRP2 produced positive results in more than 95% of *P. falciparum* bands, while ≥45 ng/mL of pLDH was required for at least 90% of pan bands to be positive.

**Conclusions:**

In active *P. falciparum* infections it is common for combination RDTs to return a positive HRP2 band combined with a negative pan-pLDH band, and when both bands are positive, often the pan band is faint. Thus active infections could be missed if the presence of a HRP2 band in the absence of a pan band is interpreted as being caused solely by persistent antigenaemia.

## Background

Antigen-detecting malaria rapid diagnostic tests (RDTs) are an important tool for fever case management and routine malaria surveillance. Available malaria RDTs can detect *Plasmodium falciparum* only, *Plasmodium vivax* only, all human *Plasmodium* species or a combination of species. Combination RDTs typically contain two test bands: a *P. falciparum*-specific test band using either bound anti-histidine rich protein 2 (HRP2) or anti-*P. falciparum* specific plasmodium lactate dehydrogenase (Pf-pLDH) monoclonal antibodies (Mabs), while the other test band typically detects either all four human *Plasmodium* species (bound anti-pan specific pLDH or anti-aldolase Mabs) or is species-specific for non-falciparum malaria (against one or more *Plasmodium* species).

Many studies have reported the performance of HRP2 and pLDH-based RDTs for detecting *P. falciparum* mainly compared to microscopy, as the reference standard [1]*.* A systematic review of 48 studies describing malaria diagnostic performance that accounted for the imperfect reference standard indicated that although performance varied by species, parasite density and immunity, overall HRP2-detecting RDTs outperformed pLDH-based RDTs and microscopy with high sensitivity and specificity for diagnosing malaria in clinical cases in endemic settings, and also asymptomatic malaria infections in endemic areas [2]. However, HRP2-detecting RDTs are unsuitable for monitoring parasite clearance following anti-malarial treatment due to the persistence of the PfHPR2 antigen in the blood for up to four or five weeks following curative treatment of an infection [1-3].

The issue of persistent antigenaemia in endemic areas has been raised as a factor leading to reduced specificity of HRP2-detecting RDTs for diagnosing acute malaria and over-estimates of malaria prevalence in community surveys [4,5]. Some studies compare HRP2-detecting RDTs to polymerase chain reaction (PCR) and/or microscopy results to distinguish between persistent antigenaemia, and acute infection; and alternatively, it has been suggested that because pLDH is metabolized after three to five days following anti-malarial therapy, that pan-pLDH test bands on combination RDTs could be used to distinguish between persistent antigenaemia (following treatment), and active infection [6]. However, this interpretation is contrary to manufacturers’ instructions for HRP2 and pan-pLDH or aldolase-based, combination RDTs, which define a *P. falciparum* infection as either a single HRP2 test line reaction or both HRP2 and pan-test line reactions. Ultimately, any modification to manufacturers’ recommendations would only be valid if it can be shown that active infections do not produce the same result (HRP2 test line positive only) as post-treatment antigenaemia.

To investigate the relationships between HRP2 and pan band positivity, the current study analyses the individual band reactivity of 18 *P. falciparum*/pan combination RDT products against samples collected from patients with active *P. falciparum* infection and without history of anti-malarial therapy in the past month. The relationship between test band positivity and antigen concentration in these samples is also described to help explain the observed results.

## Methods

In 2008, an evaluation program to assess the performance of commercially available malaria RDTs was launched by the Western Pacific Regional Office of the World Health Organization (WHO), the Special Programme for Research and Training in Tropical Diseases (TDR) and the Foundation for Innovative New Diagnostics (FIND). Across the first five rounds of product testing conducted between 2008 and 2013, this programme has assessed a total of 210 RDT products, including 63 independent combination products to detect and differentiate *P. falciparum* and non-*P. falciparum* species (hereafter referred to as Pf + pan products). Data generated during this product testing provides a unique opportunity to assess and compare the performance of RDTs as well as individual test bands on well characterized *P. falciparum* clinical samples.

The full testing protocol is described elsewhere [7]. The specific focus here in is on the results of testing each RDT product against a panel of approximately 100 *P. falciparum* wild-type parasites at 200 and 2,000 or 5,000 parasites/μL. Over the five rounds of testing a total of 157 parasite samples have been used in these wild-type panels. Each parasite sample was confirmed by PCR to be a mono-infection with *P. falciparum*, and to contain the gene encoding for the PfHRP2 protein. Patients were excluded if they had received any form of anti-malarial therapy in the four weeks preceding enrolment. Antigen levels of each sample were measured by quantitative ELISA for HRP2, pLDH and aldolase [7].

Although a total of 63 Pf + pan products have been tested by the programme, only 18 currently meet the WHO recommended criteria for procurement of malaria RDTs: products that have been tested within the last five years and have high rates of detection (>75% panel detection score) of both *P. falciparum* and *P. vivax* at 200 parasites/μL, and low false positive (<10%) and invalid (<5%) rates [8]. Of these 18 products, the large majority (15) use HRP2 as the target *P. falciparum* band antigen and pan-pLDH as the target antigen for the pan band (Table 1). Raw data for these 18 products was extracted from the full product testing results database for analysis in the current study.

**Table 1:** **Combination RDT products included in this study**

**Product**	**Manufacturer**	***P. falciparum*** **band antigen**	**Pan band antigen**	**No. Pf samples** ^**a**^
Advantage Malaria Pan + Pf Card - IR231025	J. Mitra & Co. Pvt. Ltd.	HRP2	Pan(pLDH)	100
BIOCREDIT Malaria Ag Pf/Pan (HRPII/pLDH) - C30RHA25	RapiGEN INC.	HRP2	Pan(pLDH)	100
BIONOTE MALARIA P.f.& Pan Ag Rapid Test Kit - RG19-08	Bionote,Inc.	HRP2	Pan(pLDH)	99
BioTracer™ Malaria Pf/PAN Rapid Card - 17012	Bio Focus Co., Ltd.	HRP2	Pan(pLDH)	100
CareStart™ Malaria/Pregnancy Combo - G0221 (pLDH/HRP2/HCG)	Access Bio, Inc.	HRP2	Pan(pLDH)	99
CareStart™ Malaria HRP2/pLDH (Pf/PAN) COMBO - G0131	Access Bio, Inc.	HRP2	Pan(pLDH)	100
CareStart™ Malaria pLDH 3 Line Test - G0121	Access Bio, Inc.	Pf(pLDH)	Pan(pLDH)	99
CareStart™ Malaria Screen - G0231	Access Bio, Inc.	HRP2 / Pf(pLDH)	Pan(pLDH)	99
DIAQUICK Malaria P.f/Pan Cassette - Z11200CE	DIALAB GmbH	HRP2	Pan(pLDH)	100
EzDx^TM^ Malaria Pan/Pf Rapid Test Detection kit - RK MAL 001	Advy Chemical (Affiliate of Bharat Serums & Vaccines Ltd.)	HRP2	Pan(pLDH)	100
HiSens Malaria Ag Pf/Pv (HRP2/pLDH) Card - HR2923	HBI Co., Ltd.	HRP2	Pan(pLDH)	100
Humasis Malaria P.f/Pan Antigen Test - AMAL-7025	Humasis, Co., Ltd.	HRP2	Pan(pLDH)	100
Malaria Pf/Pan One Step Rapid Test - RT 20222	Zhejiang Orient Gene Biotech Co., Ltd.	HRP2	Pan(pLDH)	100
NanoSign Malaria pf/pan Ag 3.0 - RMAP10	Bioland Ltd.	HRP2	Pan(pLDH)	98
OnSite Pf/Pan Ag Rapid Test - R0113C	CTK Biotech, Inc.	HRP2	Pan(pLDH)	100
ParaHIT - Total Ver. 1.0 (Device) - 55IC204-10	Span Diagnostics Ltd.	HRP2	Aldolase	98
SD BIOLINE Malaria Ag P.f/Pan - 05FK60/05FK63	Standard Diagnostics Inc.	HRP2	Pan(pLDH)	100
SD BIOLINE Malaria Ag P.f/Pan - 05FK66	Standard Diagnostics Inc.	HRP2	Pan(pLDH)	98

^a^Number of *P. falciparum* samples included in the wild type testing panel.

Each product was tested on each *P. falciparum* sample a total of six times; four times using the sample diluted to 200 parasites/μL and two times using the sample at 2,000 or 5,000 parasites/μL. For each test the band intensity of the control and test bands were graded on a visual scale between 0 and 4 using standard colour charts at the minimum read time recommended by the manufacturer. These results are the basis of this analysis with a band intensity of 0 being classified as negative and intensities of 1, 2, 3 or 4 being classified as positive. Any tests which returned a negative result on the control band were classified as invalid and excluded from all analysis. The overall test result was interpreted according to the manufacturer instructions:

1. a positive *P. falciparum* test band (hereafter referred to as the Pf band) was classified as a positive test for *P. falciparum*, irrespective of the result on the pan test band,
2. a positive result on the pan band when the Pf band was negative was classified as a positive test indicating a non-falciparum infection, and
3. a negative result on both test bands was classified as a negative test result indicating no *Plasmodium* infection.

Since all samples used during testing were known to contain only *P. falciparum* parasites, tests positive for a non-falciparum infection were classified as false positives (non-*P. falciparum*).

### Statistical analysis

The statistical analysis was conducted using SPSS Statistics Version 21 (IBM). For RDTs testing positive to *P. falciparum* the mean HRP2 band intensities of tests with a positive pan band were compared to those with a negative pan band using a paired t-test, with the RDT product used as the basis for pairing. Pearson’s correlation was used as the measure of association between HRP2 and pLDH antigen levels within parasite samples.

## Results

### Test and band positivity

A total of 7,160 and 3,580 tests were conducted using the 18 RDT products and *P. falciparum* samples containing 200 parasites/μL and 2,000 or 5,000 parasites/μL, respectively. These tests yielded 7,146 and 3,574 valid RDT results for the low and high parasite concentrations, respectively.

**Figure 1:**
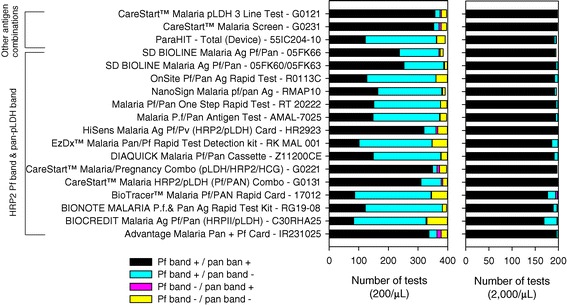
**Performance of individual test bands of RDT products when tested using wild-type** ***P. falciparum*** **parasites at densities of 200 parasites/μL and 2,000 parasites/μL.**

When tested against 200 parasites/μL, 92.3% of the tests returned a positive result for *P. falciparum*, 0.9% returned a false positive result for a non-falciparum infection and 6.7% of tests were negative. Of the tests that were positive for *P. falciparum* infection using the low parasite density samples, 57.1% were positive on both the Pf and pan bands, while 42.9% were positive on the Pf band only. The proportion of tests positive for *P. falciparum* varied between individual RDT products (range 81.8% to 96.9%, Figure 1). There was also considerable product variability in the proportion of positive RDTs with a positive pan band (range 25.0% to 95.9%, Figure 1).

When tested using parasites at the higher concentration of 2,000 or 5,000 parasites/μL, 99.7% of tests were positive for *P. falciparum* with 96.7% of these tests returning a positive Pf and pan band. False positive results for a non-falciparum infection occurred in 0.2% of tests, while 0.1% of tests were negative for *Plasmodium*.

### Band intensities

The mean band intensity for tests positive on the Pf band at 200 parasites/μL was 2.47 (standard deviation (sd) 0.96) for the HRP2-detecting Pf bands and 2.84 (sd 1.03) for PfpLDH-detecting Pf bands. In contrast, the mean intensity for positive pan bands was lower at 1.50 (sd 0.78), with 65% of tests having a pan band intensity of 1. All band intensities increased when products were tested using samples containing 2,000 or 5,000 parasites/μL; mean band intensities were 3.68 (sd 0.63), 3.91 (sd 0.32) and 2.43 (sd 0.98) for the HRP2-detecting Pf bands, PfpLDH-detecting Pf bands and the pan bands, respectively. At this higher parasite density 16.1%, 42.0%, 23.5% and 18.4% of RDTs returning positive results on both test bands had pan band intensities of 1, 2, 3 and 4, respectively.

A direct comparison of the pan and Pf band intensities of individual tests at the lower parasite density revealed that in 0.9%, 12.7% and 86.4% of tests the pan band had a greater, the same, or less intensity than the Pf band, respectively. For the 5,720 tests which had a higher Pf band intensity compared to the pan band intensity, the difference in band intensities was one unit for 39.2% of tests, two units in 37.8% of tests and three units in 19.1% of tests.

**Figure 2:**
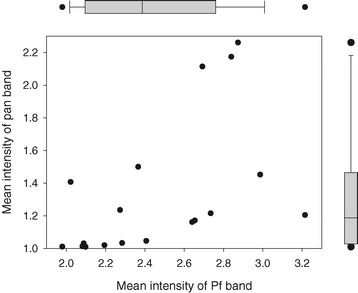
**Mean band intensities for 18 RDT products when tested against wild-type** ***P. falciparum*** **parasites at 200 parasites/μL.** The overall distribution of the Pf and pan band intensity is illustrated by the corresponding box plot; box extends from first to third quartile, with line indicating median.

The mean band intensities for positive RDTs with HRP2-detecting bands varied by product with values ranging from 1.98 (BioTracer™ Malaria Pf/PAN Rapid Card - 17012) to 3.21 (BIONOTE MALARIA P.f.& Pan Ag Rapid Test Kit - RG19-08) when tested against 200 parasites/μL (Figure 2). Product means were approximately normally distributed around the overall mean of 2.47. The mean intensities for positive pan bands also varied by product ranging from 1.01 (EzDx RK MAL 001) to 2.26 (Carestart G0231), but the distribution was positively skewed with 50% of the products having a mean band intensity less than 1.19 and only three products (Carestart G0221, Carestart G0121 and Carestart G0231) having means greater than 2.1.

**Figure 3:**
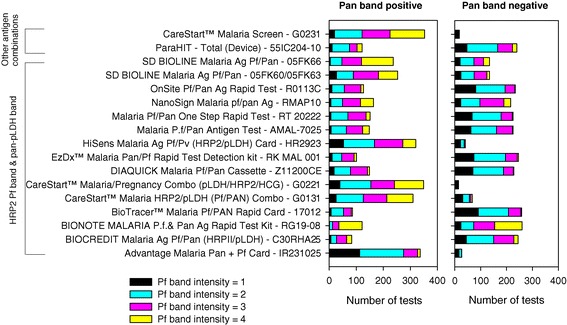
**Distribution of Pf band intensities for HRP2-detecting combination RDTs returning a positive result against samples containing 200** ***P. falciparum*** **parasites/μL.** Tests are grouped based on whether the pan band was positive (left) or negative (right) in the individual tests.

A more focused analysis of band intensity was conducted for RDTs with a HRP2-detecting Pf band using the results of testing with samples containing 200 parasites/μL. For each product the mean and distribution of the Pf band intensity was calculated for tests that were positive on both the Pf and pan bands and those that were positive only on the Pf band (Figure 3). Comparison of the mean values revealed that on average, the Pf band intensity for a product was 0.73 units (95% CI: 0.53-0.93 units) higher when both the Pf and pan bands were positive (mean = 2.71, sd 0.39) compared to those where only the Pf band was positive (mean = 1.97, sd 0.48) (paired t-test, t_16_ = −7.63, p < 0.001).

### Relationship of antigen concentrations in wild-type *Plasmodium falciparum* samples and positivity of test bands

**Figure 4:**
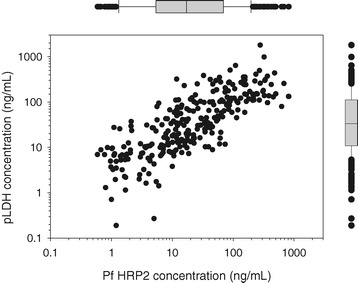
**PfHRP2 and pan-pLDH concentrations of wild type** ***P. falciparum*** **samples (at all dilutions) used for testing RDTs.** The overall distribution of the HRP2 and pLDH concentrations is illustrated by the corresponding box plot; box extends from first to third quartile, with line indicating median.

The *P. falciparum* samples used in the testing of the 18 RDTs had varying antigen concentrations. When the data for both the low parasite density (200 p/ul) and the high density (2,000 or 5,000 p/ul) samples were pooled, the HRP2 concentrations ranged from 0.62 ng/mL to 820.2 ng/mL, while pLDH concentrations ranged from 0.19 ng/mL to 1,800 ng/mL (Figure 4). The HRP2 and pLDH concentrations within individual samples were significantly correlated (r = 0.45, p < 0.001).

**Figure 5:**
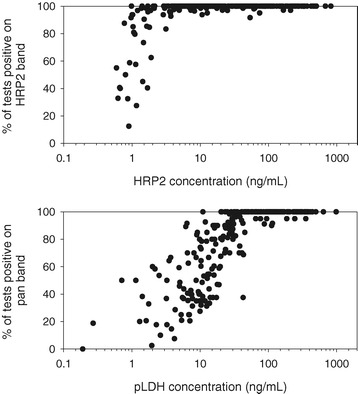
**Relationship between antigen concentration and RDT test band positivity for HRP2 (top) and pan-pLDH (bottom).** Only samples used to test 10 or more individual RDTs are displayed.

There was a clear relationship between antigen concentration and band positivity rate (Figure 5). With the exception of two samples, HRP2 concentrations of 4 ng/mL or greater produced positive results in over 95% of HRP2-detecting test bands. Below this threshold the test band positivity was highly variable showing a rapid decline with concentration.

A similar pattern was observed for pLDH and pLDH-detecting test bands; pLDH concentrations above 45 ng/mL returned positive pLDH test bands in over 90% of tests. Below this threshold the positivity of the pLDH test bands declined with concentration (Figure 5).

## Discussion

The HRP2 antigen is known to persist within the circulation following curative treatment and this has led to the suggestion that HRP2-detecting RDTs have reduced specificity to detect active malaria infection in moderate to high transmission areas. On the other hand, pLDH is metabolized more quickly and, therefore, RDTs detecting this enzyme are expected to revert to negative more quickly following malaria treatment. Therefore, a tempting strategy to differentiate past, treated infections from current infections is to use combined HRP2 and pan detecting RDTs, or RDTs that contain separate HRP2 and Pf-pLDH test bands. While this approach initially sounds reasonable in circumstances where a recent infection has occurred [9], its general application to clinical management of fever cases is subject to discussion. Hawkes *et al.* [6] conclude that requiring positivity of both the HRP2 and pLDH bands in a combination RDT can improve diagnostic specificity for falciparum malaria in a sub-Saharan African context, by excluding false positive HRP2 results due to persistent antigenaemia. This conclusion was reached after comparing RDT results to microscopy in a sample of children under five years of age hospitalized for febrile illness. While this suggestion may be appropriate for selection of individuals for clinical trials, the extension of this to clinical diagnosis or management is questionable since this conclusion is based on the assumption that all RDTs that are HRP2-positive but pLDH-negative represent persistent antigenaemia. Underpinning this assumption is the premise that all current infections produce positive results on both the HRP2 and pLDH bands of the RDTs, an assertion that has not been tested previously in a systematic way for multiple RDT products*.*

To address this gap this study analysed data generated during the WHO-FIND product testing programme for malaria RDTs with a specific focus on the reactivity of individual test bands in 18 combination malaria RDTs that meet the current WHO recommended procurement criteria. Seventeen of these products used PfHPR2 for the detection of *P. falciparum.* During WHO-FIND product testing, these 18 products were able to consistently detect >75% of the wildtype *P. falciparum* and *P. vivax* samples at a dilution of 200 parasites/μL with low false positive rates or incorrect species identification. Although this study focused on the detection of *P. falciparum,* the RDTs selected for inclusion in this analysis were purposely restricted to those meeting the criteria for both *P. falciparum* and *P. vivax* detection to be assured that the pan test bands were performing well when interpreted according to the manufacturer’s instructions. This removes the possibility that the results observed in this study were caused by a dysfunctional pan band.

The results of the current analysis indicate that there is a difference in the sensitivity of the HRP2-detecting test bands and pan-pLDH test bands in the detection of active infection. At low parasite densities it was observed that the HRP2-detecting band returned a positive result in the absence of a positive pan band in over 40% of positive tests, with this percentage being product specific. This trend was less evident at higher parasite densities where both bands tended to be positive simultaneously. This result matches that previously reported for the SD BIOLINE Malaria Ag P.f/Pan (catalogue number 05FK60, Standard Diagnostics Inc., South Korea) product where the proportion of tests positive on both the HPRP2 and pLDH bands progressively increased with parasite density from 6.7% at <100 parasites/μL to 98.5% at >1000 parasites/μL [10]. Similar patterns were also reported for the SD Malaria Antigen P.f (catalogue number 05FK90, Standard Diagnostics Inc. South Korea) product [11].

The analysis also revealed that even when both the pan and *P. falciparum* bands were positive, the band intensity of the pan band was generally lower than the Pf band, irrespective of the parasite density. At the lower parasite density of 200 parasites/μL, 65% of the positive pan bands had an intensity of 1. This proportion reduced to 16% when samples contained 2,000 or 5,000 parasites/μL. Thus even when the pan band is positive, it is often faint. The RDT testing conducted for the WHO-FIND product testing programme occurs at the CDC in ideal conditions which assists with the identification of these weak positive results. However faint bands are often difficult to see and may well be missed by health workers working in reduced lighting conditions or with reduced visual acuity [12,13]. This would inflate the proportion of RDTs having a positive HRP2 band and negative pan band.

There are several possible reasons for the observed differences in positivity and intensity of HRP2 Pf bands compared to pLDH pan bands. First, the relative abundance of HRP2 and pLDH may differ within parasites. The samples used within the current study had wide but similar ranges of concentrations for HRP2 and pLDH and there was a significant positive correlation between the two antigen concentrations. Hence it does not appear that gross differences in antigen concentration were the cause of the disparity in test band performance against *P. falciparum.*

The second possible reason is related to differences in avidity of the antigen for binding the antibodies bound to the RDT test lines. The current results indicate that approximately 4 ng/mL of HRP2 is required to obtain a positive HRP2 band in over 95% of tests, compared to over 45 ng/mL for pan-pLDH. This difference in concentration aligns with the HRP2 antigen having multiple binding epitopes due to its repeat structure, compared to pLDH, which is a single epitope. Lee *et al.* [14] reported the most frequent HPR2 motifs occurred within the HRP2 sequence with a frequency of 8–25, depending on the motif and specific sequence. This frequency aligns approximately with the differences in threshold concentration observed here. Thus, it appears that the difference in antibody-binding avidity between HRP2 and pLDH may be a cause of differences in sensitivity of the respective test bands. It is unlikely that the ordering of the bands on the strip accounts for diminished performance of the pan test bands because for all except two tests included in this analysis the pan test band is the farthest from the origin and this position is advantageous as the flow rate at which the analyte passes the capture reagent line is slower; and the effective concentration of analyte in the sample is higher [15].

Many studies have been conducted to assess the performance of specific malaria RDTs in different settings [1]. However few have directly compared HRP2-detecting RDTs to pLDH-detecting RDTs, and examined potential reasons for the differences in the two types of tests. A longitudinal study in Uganda identified that HRP2-detecting RDTs provide better detection of parasites at low densities compared to pLDH-detecting RDTs, but have lower specificity due to the slower clearance of HRP2 antigenaemia from the blood circulation [16]. Differences have also been observed in performance between regions with different transmission intensities, and this difference was attributed to the superior ability of HRP2-detecting RDTs over pLDH-detecting RDTs to detect sub-patent parasitaemia [17]. These results, although obtained by comparing different RDTs that both detect *P. falciparum,* appear relevant for the combination tests with antibodies against these two antigens. It would be possible to distinguish between persistent HRP2 antigenaemia and viable falciparum parasitaemia in a given blood sample by comparing the results of an HRP2-based RDT with a separate, equally well-performing RDT containing a falciparum pLDH band, but that is not a viable proposition for field use.

Therefore, the problem remains of how to clinically manage a febrile patient with a history of recent anti-malarial treatment who returns a positive HRP2 band, but a negative pLDH band on a combination RDT. This can result from persistent antigenaemia or malaria re-infection or recrudescence (treatment failure). Treatment failure may result from drug resistance or inadequate exposure to the drug due to sub-optimal dosing, poor adherence, vomiting, unusual pharmacokinetics in an individual or substandard medicines. It is important to determine from the patient’s history whether he or she vomited the previous treatment or did not complete the full course of treatment. These cases need to be treated again with the artemisinin-combination therapy (ACT) recommended as first-line in the area.

If the patient’s history reveals that he/she has taken the full and correctly dosed treatment course, the possibility of true treatment failure can only be excluded by referring the patient to a facility with good quality microscopy. Referral may be necessary anyway to obtain second-line treatment. In individual patients, it may not be possible to distinguish recrudescence from re-infection, although lack of resolution of fever and parasitaemia (on microscopy) or their recurrence within four weeks of treatment are considered failures of treatment with the currently recommended ACT. For these cases the recommended second-line treatment is an alternative ACT known to be effective in the region. In addition to the above guidance, in all cases the health provider should always consider other diagnoses and follow closely for a clinical response.

Recurrence of fever and parasitaemia more than four weeks after treatment may be due to either recrudescence or a new infection. The distinction can only be made by genotyping of parasites from the initial and the recurrent infections. As parasite genotyping is not routinely used in patient management, then all presumed treatment failures after four weeks of initial treatment should be considered new infections and be treated with the first-line ACT.

Ultimately, the results from this study clearly show that in the setting of active (untreated) malaria infection, it is common for HRP2/pan-pLDH combination tests to return a positive HRP2 band combined with a negative pan-pLDH band at low parasite densities, and when both bands are positive, often the pan band is faint even at densities of 2,000 parasites/μL. Therefore it would be dangerous to interpret the presence of a HRP2 band in the absence of a pan band as being caused solely by persistent antigenaemia in a clinical setting. Only when the sensitivity of the pLDH-detecting pan band is improved to have the comparable reactivity as the HRP2 band for detecting *P. falciparum* could persistent antigenaemia be confidently attributed as the cause of HRP2-positive, pan-negative RDT results.
